# The Hospital of Tomorrow Case Study: Multidisciplinarity, Inclusiveness and Holistic Approaches to Foster Innovation in Complex Organizations

**DOI:** 10.34172/ijhpm.2023.7330

**Published:** 2023-02-28

**Authors:** Francesca Gorla, Anja Borojevic, Chiara Gibertoni, Lorena Landi, Marco Storchi, Luca Fontana, Jetri Regmi, Barbara Burmen, Anna Silenzi

**Affiliations:** ^1^World Health Organization, Headquarters, Geneva, Switzerland.; ^2^IRCCS Azienda Ospedaliero-Universitaria di Bologna, Policlinico di Sant’Orsola, Bologna, Italy.; ^3^World Health Organization, Regional Office for Europe, Copenhagen, Denmark.; ^4^James Lind Institute, Geneva, Switzerland.

**Keywords:** Project Management, Participatory Process, Groups Empowerment, Hospital Management, Health System, Hospital Organization

## Abstract

**Background:** This case study describes and analyses an action research initiative undertaken by management, staff and WHO at the St. Orsola-Malpighi Polyclinic in Bologna, Italy. The initiative utilised staff engagement approaches developed during the COVID-19 pandemic to rethink and reshape future development plans. The initiative provides a ‘how-to’ case study for complex health facilities on ways to create similar multisectoral, inclusive and holistic processes in planning structural, functional and organizational solutions for their ‘hospitals of tomorrow’.

**Methods:** The case study utilised an action research approach coordinated by a team of WHO facilitators in close collaboration with the Board of Hospital Directors. Heterogeneous and multidisciplinary working groups were created, with members from different levels of the hospital staff. In the context of facilitated group meetings held weekly over a one-year period, participants were asked to review topics of interest to future plans of the hospital and make recommendations on effective/innovative ways of addressing these in the short and long term. Working groups focused on different challenges.

**Results:** The initiative was successful in creating and sustaining broad staff engagement in the future planning processes. 80% groups maintained high staff participation throughout the entire project year. Participating staff reported enhanced communication and cooperation between departments represented in different groups. 87% of the proposed plans suggested by the working groups were approved by the Board for implementation.

**Conclusion:** Key factors contributing to the high approval rate of plans, strong engagement record of staff and enhanced cooperation between involved departments; included: multisectoral/cross hierarchal staff involvement, group attention to defining time-bound contextual goals, flexible implementation monitoring approaches, personnel skills and profiles of participants, direct and open communication at all levels and times, member commitment and clear exit strategy. The case study is presented as a model to stimulate similar actions in other complex health care facilities.

## Background

**Key Messages**

** Implications for policy makers**

‘Future’ planning can be enhanced, and made more implementable, by action research processes that:

- Involve multi-disciplinary staff groups from different hierarchical levels within the organization.
- Create groups that allow for shared responsibility, commitment and ownership of participant generated innovative solutions.
- Ensure continuity of planning and projects and ensure that ‘model’ projects are integrated into normal planning and budgeting approaches.
- Focus on building resilience that go beyond emergency situations.

** Implications for the public**

This action research case study provides a model of working that can enhance staff engagement in developing innovative solutions for future planning, and participants’ action competence and influence in shaping future plans and actions. This model aims to allow replication in other complex health settings which highlights the importance of communication amongst different departments, commitment and a shared vision of goals derived from both bottom-up and top-down approaches, ensuring that projects are kept within the groups’ memories and that changes in management, staff do not affect the objectives achievement. Communication in complex organizations is key to successful implementation of projects and solutions, especially in emergencies and fast changing needs and the success of the development of various projects and solutions is ensured by shared responsibilities that allow horizontal democracy to take place.

Italy was one of the ﬁrst European countries to report COVID-19 cases (January 2020) and community-based transmission (February 2020).^1,2^ There was an unprecedented overload of the health system, with a rapid increase in the number of severely ill patients needing emergency and intensive care. Health facilities were challenged to find effective ways of addressing COVID-19 infected patient care needs whilst maintaining safe environments for other patients, visitors and staff; eg, to find ways to provide well demarcated paths to separate the infected from non-infected. In March 2020, the Italian Ministry of Health introduced legislative decrees regarding the need for ongoing changes to enhance hospitals’ flexibility and capacity to deal with the pandemic.^3-6^ Health facilities began to respond. Some turned to WHO and other agencies for assistance in adapting their hospital capacities, organization and infrastructures to address the needs of their growing number of COVID-19 cases. In May 2020, for example, the IRCCS Azienda Ospedaliero-Universitaria di Bologna, Policlinico di Sant’Orsola, Italy requested and started to receive on-site World Health Organization (WHO) assistance for the adaptation of their emergency room and other departments.

 Building on this experience, the Hospital, in May 2020, asked WHO to help them develop and implement a working model that would involve a broader array of staff in developing innovative solutions to be applied in their longer-term planning and renovation of several pavilions and what came to be called their ‘Hospital of Tomorrow.’ The expressed aim here was to use their COVID-19 pandemic experiences to help make their facilities^7^ more resilient to future challenges. This request shifted the WHO focus from consultation to action research aimed at facilitating the co-production of future plans. This case study describes the process and outcomes of this action research initiative and its potential use as a model for other complex heath care facilities.

###  The Facility

 IRCCS Azienda Ospedaliero-Universitaria di Bologna, Policlinico di Sant’Orsola, Italy was founded in 1592 in Bologna, Italy. It is a university and public hospital, IRCCS (Scientific Institute for Research, Hospitalization and Healthcare). It has 7 departments and includes 91 operative units. It is equipped with 1758 beds and staffed by 5355 employees including university researchers and physicians.^17^ The grounds occupy an area of approximately 0.22 km^2^ with its Operative Units distributed among 31 pavilions. An estimated 20 000 people including staff, students, university teachers, patients, visitors and suppliers, are present on the hospital grounds on any given day.^8^

## Methods

 We conducted a qualitative case study, identified as most adapt methodology to capture the fluidity of the case, including temporal changes, as well as explore the contextual conditions in complex healthcare innovations.^9,10^ This specific facility, already engaged in a collaboration with WHO, requested support to implement a working model that could involve staff in finding innovative solutions to be applied to the renovation of several pavilions. A case study was used as methodology to be able to describe this process and develop the requested work model to replicate for other facilities.

 Problems statements and strategic areas of intervention were initially defined with participative discussion in collaboration with the Board of Hospital Directors. In order to define methods to address key areas of work, a scoping review of literature was conducted, and key challenges were therefore translated in multidisciplinary and heterogeneous working groups with committed members from the hospital staff of different hierarchy levels and WHO facilitators.

 Participants to the working groups were chosen by the Board of Directors based on their professional competencies, interest in the topic and the correlation between the topic and their field of work. Specific competences were valued important to contribute to the overall knowledge of the covered topic both to achieve the objectives and to share this knowledge with the rest of the participants, increasing the collaboration and communication between different departments. Participants were also included when the correlation between the discussed topic outcomes had an impact on their department or field of work, even if the competencies on the subject were not required by their professional profile.

 Theoretical frameworks were designed to enable problems analysis and formulate hypothesis. In order to keep track of the process and investigate critical success factors, data were collected and collated through meeting reports and surveys. Impacts indicators were developed through group discussions of facilitators and Board of Directors.

 This study seeks to contribute to academic literature on multidisciplinary engagement in complex organizations by addressing two research questions:

1. What model of work to apply in complex organizations, seeking for innovative and bespoke solutions, ensure multidisciplinary engagement and participatory methods in the healthcare context?
2. Which methods and strategies ensure process resilience during disruptive events such as emergencies, role changes, and management changes?

We conducted a scoping review to investigate the role of employees and participatory processes in co-designing innovative solutions concerning several key aspects – structural, procedural, managerial- that characterize hospitals. The review was done focusing on healthcare facilities, being these a one-of-a-kind complex structure.

 Different experiences describing successful outcomes with participatory methods involving healthcare professionals along with technical and administrative staff, regardless the hospital hierarchy, has been reviewed, setting the basis for the methodology proposed in the presented case study.

 Numerous research works underline that large participation of committed and empowered^11,12^ hospital employees involved in co-design processes, do play a role in fostering innovation within the healthcare sector by enhancing intra-organizational collaborative approach.^11,13-15^ These factors also contribute to improving managerial tools and organizational performances by striving for the shared goals of employees and managers.^16,17^ Furthermore, participatory approaches foster translation of research findings into practice.^18^

 The literature suggests that successful groups are heterogeneous, interdisciplinary, presenting cross-hierarchy participants,^11,13,14^ combining formal and informal working methods and communication processes^13,17^ to work on problem setting and problem solving.^14^

 Project management professionalization, by strong project management processes and instruments,^15,19^ and support provided by key stakeholders,^12^ which prevent process segmentation and providing a clear setting for running and managing meetings,^14^ increases project success and therefore hospital innovativeness.

 Thus, the mentioned aspects have been included in the case study hereafter presented. The case study aims to contribute to the literature on how multidisciplinarity, inclusiveness and holistic approaches can foster innovation in complex organizations such as healthcare facilities, which has not been studied in details.^11,12,15^

###  The Case Study Presentation

####  The Approach

**Figure 1:**
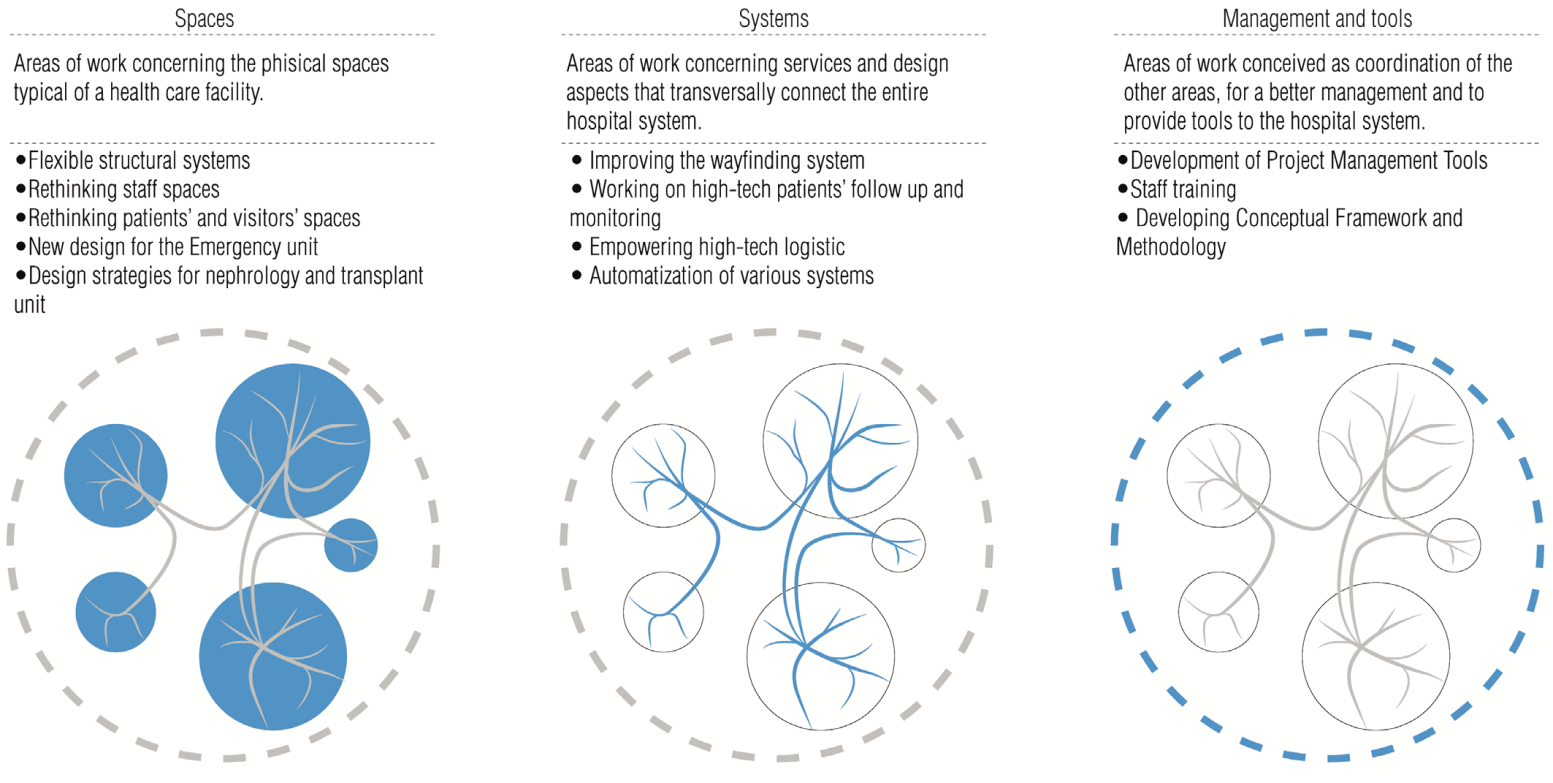
Project Conceptual Framework.

 The first step of the project implementation included a preliminary participative phase where the Board of Hospital Directors, together with the WHO team, extensively analyzed the entire hospital organization, clarified the existing hierarchical system and investigated areas of strength and weaknesses.^15^ The aim of this process was to identify broad goals and sub-goals, and to define a clear rational initial and subsequent action plan. The team then consolidated this information into a logical framework that addressed work areas focused on the hospital’s physical spaces, hospital services and design aspects and areas focused on the coordination and management of other areas or processes. Later the framework was reviewed and adapted to new emerging needs (Figure 1).

####  The Hospital Working Groups

 Hospital working groups were initially created according to the strategic vision of the Board of Hospital Directors. Members were selected on the basis of the projects’ affinity to their professional profile and competencies and in agreement with their direct supervisor and Board of Hospital Directors. Groups were meant to answer to previously detected needs and issues and therefore enact the completed logical framework. Group members and their representatives were initially selected by the Board of Hospital Directors to mirror macro areas of work and involve all the stakeholders required for specific development processes. Working groups consisted of professionals from different departments who had different roles. Cross-functional and interdepartmental teams would tackle issues based on a mix of transversal skills and expertise: analyzing multifaceted topics and brand-new challenges from multiple perspectives would have allowed to take advantage of a positive combination of competences. Groups were comprised of technical, administrative and health workers at different levels of the organizational hierarchy, such as directors, middle managers and regular employees with equal gender balance. During the project meetings, invited speakers from outside and within the hospital organization attended on an ad hoc basis to support groups with specific expertise. The nine groups established between September 2020 and August 2021 are listed below:

 High tech logistics: this work area refers to a complex and dynamic way to approach and coordinate logistics by looking for solutions to improve accuracy, efficiency and effectiveness. State-of-the-art logistics best practices include strengthening elements such as dematerialization and traceability to improve time efficiency, smooth processes and maximize positive outcomes.

 Wayfinding: wayfinding is a key work area for such complex facility. This group’s mission was to improve the wayfinding within the St. Orsola compound to enhance path’s clarity to ensure patients,’ visitors’ and staff autonomy and safety. Other services related to patients’ reception were also addressed.

 Staff spaces: this working area is designed to broadly focus on spaces for staff in administrative and medical areas. They objective of the working group was to find solutions that would take into account existing innovations, psychosocial and environmental aspects, as well as the safety and well-being of the staff.

 Operational support system: this working group focused primarily on the empowerment of the staff and ultimately the hospital through the use of specific tools and project management software.

 Patients’ and visitors’ spaces: this working group looked at innovative solutions that could potentially be utilized as a hospital standard to plan for future hospital design and to re-think patients’ and visitors’ spaces based on the lessons learned from the ongoing COVID-19 pandemic.

 Users’ reception: this group focused on the improving hospital users’ reception services, starting from an analysis of the frequent claims expressed through formal reports by visitors and patients on the way accesses are managed by stakeholders in charge. This enabled the group to begin developing solutions for all identified problems.

 Emergency unit: this group attended ad hoc meetings to work on the design of the new layout of the emergency department.

 Conceptual framework and methodology: the objective of this group was to work towards systematizing work processes and problem-solving tools in different scenarios, focusing mostly on COVID-19 related issues such as vaccination campaign, and the preparation and use of COVID-19 wards.

 Telemedicine: the group, incepted under Hospital Directors mandate, worked mainly by interviewing the hospital staff on practices of telemedicine undertaken by the different departments and assessing all the technologies available and the solutions implementable.

 Each group’s strategic areas of work were closely linked to the dynamic hospital environment. The goals set by each of the working groups were a combination of inputs initially provided by the Board of Directors and inputs proposed by the group participants. Later, as the topics were discussed and gradually adapted to the emerging needs of the hospital, some working groups were absorbed by others or restructured and other groups were newly created.

####  The Groups’ Way of Working

 The need for staff to address high levels of change and uncertainty underlined the importance of focusing on communication streams and staff interaction within the hospital. The groups identified the need to re-think and promote innovative strategies and concerted actions to foster a positive environment while simultaneously improving organizational standards and procedures. Therefore, participatory processes and synergies between departments and levels of responsibility ensured efficiency, innovation and a cohesive response to the dynamic environment.^11^ Subsequently, in line with directions given by the Board of Hospital Directors and through decision-making processes based on the examination of strategic areas of intervention, the groups conducted an initial analysis about what activities to prioritize: the hospital’s strengths and issues were mapped and investigated by groups’ members themselves, who collected information starting from what turned out to be crucial in their daily working life. Later on, as the project benefited from an increasing flexibility in participants’ recruitment: professionals were asked to permanently join the groups according to emerging needs and dynamics, or on ad hoc basis.

 Individual group meetings were held weekly; group representatives (Focal Points) also met on a weekly basis to liaise between all groups and monitor the processes. Focal Points reported progress and updates from each group, criticalities and needs to the Focal Points’ group. The Focal Point’s group, being constituted of representatives from all groups, was originally created based on the input from the Board of Hospital Directors.

 Groups were introduced to thinking processes that approached topics and goals from multiple perspectives and in all aspects. Processes such as brainstorming and mind mapping, techniques as Six Thinking Hats (De Bono), SWOT (Strengths, Weaknesses, Opportunities, and Threats) analysis and tools as Mentimeter or Padlet were used by the WHO team to support projects’ analysis and implementation. Facilitators encouraged professionals to list and prioritize goals as short, medium, long-term goals.

 The mixed top-down and bottom-up approach, that took advantage of inputs from both the Board of Hospital Directors and group members, facilitated a deep analysis of the context. While the Board highlighted broad and strategic areas requiring potential interventions, groups identified minor challenges rooted in the daily hospital environment. Moreover, the staff drawn from a variety of backgrounds who were also equipped with different skills, had a multifaceted approach to topics, initially related to the project and gradually connected to the entire hospital environment. Because topics were transversally linked to different departments, cross communication, information exchange and dissemination of a multidisciplinary approach to other hospital units/departments was promoted.

 The process in this project were implemented as a pilot project aimed to investigate the efficacy of the methods introduced and their potential scalability. Later, budget related issues were defined as the processes developed.

####  The Projects’ External Facilitators

 The WHO team structured a robust framework to encourage commitment, engagement and project ownership by hospital staff. WHO team served as processes’ facilitator, taking care of administrative tasks as scheduling meetings, preparing agendas and writing meeting reports, and by supporting the groups’ work in defining priorities, setting goals, research and consequent implementation process and, transversally creating a strong narrative thread between the groups themselves and the work they were carrying on.^20^ Moreover, being an external actor, the WHO team could observe the dynamics between working groups’ members, facilitate goals’ definition and tasks implementation, keep track of decisions, stakeholders involved and responsibilities, and promote an unconstrained analysis of issues, criticalities and potentials.

 To further enrich the process, external experts, from St. Orsola or Emilia Romagna Region network, and téchne^21^ – the technical science for health network- members took part to the process. Through téchne’s contribution, innovative inputs to the issues raised during the project implementation were provided to the groups: this contribution resulted in the identification of non-standard solutions for potential implementation.

 The interrelationships with external stakeholders, expanded dialogue spaces and a network of competencies by taking advantage of different skills and expertise, widened the range of action and promoted the projects’ technical specificity.

###  Data Collection

 Project processes and outcomes were assessed using three main tools: indicators, surveys and a SWOT analysis.

####  Indicators

 To monitor the project, the following indicators were assessed:

 (1) Groups’ composition and attendance:

- Groups’ multidisciplinarity/heterogeneity was defined as having at least three departments in a single task force.
- Horizontal democracy as measured by assessing the group’s composition in terms of the proportion of directors, middle management and employees. If one member of each category was present in each group, horizontal democracy was achieved.
- The average scheduled and ad hoc meetings attended by each participant and by each department over a 10 months’ period.
- Participants’ drop out assessed the number of participants who attended less than 30% of all meetings.

(2) Communication amongst stakeholders:

- The creation of task forces that had 2 or more departments;
- Changes in communication between departments;
- Changes in communication between the hospital employees, including middle management and the Board of Hospital Directors; and
- Initiation of new collaborative initiatives.

(3) Goals’ definition:

- Goals’ identification;
- The number of projects approved by the Board of Hospital Directors; and
- The approved goals attained and the number not attained and reasons why.

(4) Budget and timelines definition:

- Timelines associated to projects
- Projects assigned to specific budget areas.

####  Hospital Surveys 

 Three surveys were delivered in November 2020, February 2021 and June 2021, the target population consisted of all participants of the working groups.

 The first two surveys contained mainly qualitative questions that assessed participant’s satisfaction and their commitment based on the group’s composition, goals and methods.

 The third survey had more specific qualitative and quantitative questions which covered satisfaction with the overall process (methods, feeling about innovation, skills and competences acquired, company’s improvement due to the process among others) and input collection. Participants were asked to provide scores or opinions on:

- Goals and expectations: were goals clear, innovative and achievable.
- Attendance and effort required: how much effort professionals put in the project and whether they felt final outcomes were in line with it.
- Methods and tools: if these were clear from the beginning, whether and how they were useful, and which tools turned out to be more useful than others. Multidisciplinarity was also considered as an approach used during project implementation.
- Outcomes: if specific changes (positive or negative) were detected and whether the project fostered the acquisition of new skills.
- Global process: which project element proved most interesting and whether they wanted project implementation to continue beyond the survey date.

####  SWOT Analysis

 The team of external facilitators analyzed the project’s strengths, weaknesses, opportunities, and threats based on their experiences in participating in the project and changes that had been made in the hospital.^22^

## Results

###  Groups’ Composition

 Heterogeneity was ensured across various aspects of groups’ composition. The groups consisted of professionals from different areas of work: 5 to 10 departments were involved in each team. Members with professional profiles such as technical, administrative medical staff attended each session: administrative staff was the most represented category (48%), while medical staff and technical staff each contributed a quarter of the hospital staff categories represented. Horizontal democracy was promoted and achieved by ensuring that all levels in the organizational hierarchy were equally present and had a say in contributing, proposing solutions and making decisions. About 50% consisted of managers and the other 50% consisted of regular employees. The participants were also gender balanced: 58% female employees and 42% male employees. Only one working group did not achieve the goal of horizontal democracy, the conceptual framework and methodology which consisted entirely of Directors and did not include the operational part represented in other groups.

###  Meeting Attendance

 Over the ten months of working groups’ meetings, the average of attendance was 68%: five out of eight groups showed an attendance rate (60% and 70%), two groups had an attendance of between 70% and 80% and one group had an average of 80%. The overall group members’ drop-out rate was 14%.

###  Communication Among Stakeholders

 The project processes improved communication amongst departments, multidisciplinarity was also achieved in their composition and in the way group members managed content and tasks. Among the groups, six out of eight teams were divided into task forces of staff from different departments. Interdisciplinary task forces were made up of at least three areas of work.

 Employee interaction was encouraged through a variety of channels, including participation in regular meetings, face to face meetings autonomously organized by task forces and email exchanges.

 In addition, communication was enforced by scheduling meetings with the Board of Hospital Directors. From October 2020, four plenary meetings were held between the working groups and the Board of Hospital Directors to promote dialogue and present project’s achievements to foster the involvement of an increasing number of responsibility levels within the hospital organization and share inputs and guidelines for future steps. All group members were always invited to all meetings to constantly be part of the global process.

###  Goals and Achievements

 In an initial brainstorming phase moderated by the WHO team, each team identified one to six objectives. A total of 31 hospital goals were set by all teams. During the fourth plenary meeting with the Board of Hospital Directors and the two Boards for Innovation, 87% (27) of the initial set goals were positively rated by the Board to proceed: 4 goals (13%) were currently on hold or abandoned because they required further analysis by the teams or the Board.

**Figure 2:**
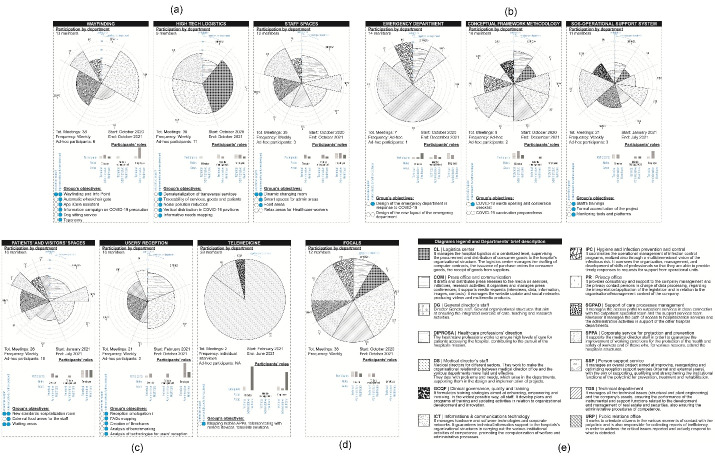
(a-e) Group composition, Meeting Attendance and Goal Attainment. Panel ‘e’ shows legend and department brief description.

 To incorporate the approved goals into the regular hospital operational lines, 15 out of the 27 approved goals (56%) and their related projects were assigned by the Directors to managers to proceed with the next steps. 10 out of the 27 goals which were positively evaluated (37%) were currently under implementation or would be implemented within the next three months; while the remaining 2 (7%) were still being analysed by the groups and require a broad investigative process (Figure 2a-2e).

###  Budget and Timeline Definitions

 Due to project framework and its development from an initial brainstorming phase and a subsequent process of next steps concrete timelines were roughly defined. Professionals tended to focus on weekly tasks and deadlines rather than to longer timelines. In addition, individual projects were not specifically structured with clear timeframes and steps.

 Since the goals defined in the first months of the process mainly related to new areas of investigation and the process itself was detached from the standard hospital operational lines, an initial stand-by situation was detected. Projects were initially not assigned to defined departments, and budget lines for consequent implementation were also not clear. Once a fair progress and a sufficient level of analysis were achieved by assigning the 15 projects to Managers responsible of their implementation, the Board of Hospital Directors specified the budget lines to be used for each activity.

###  Surveys and Results

**Figure 3:**
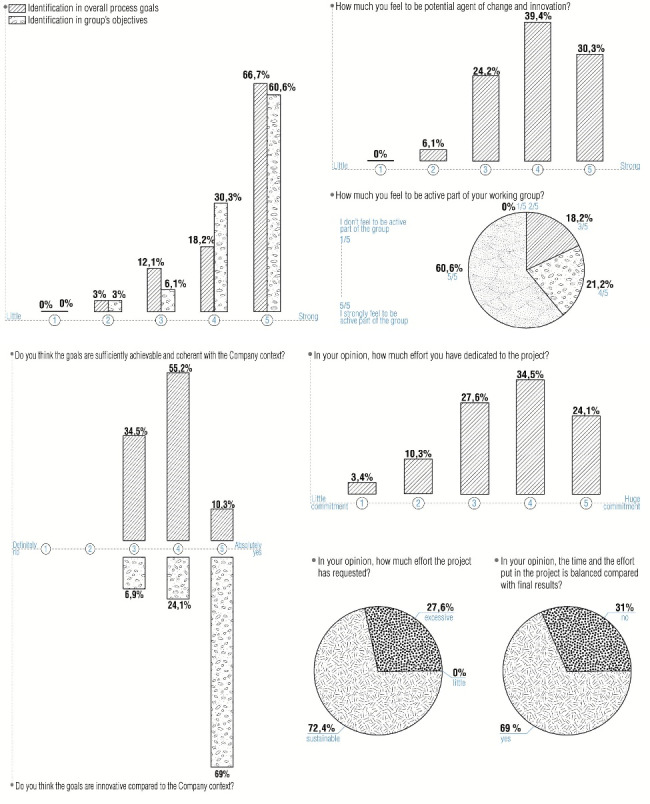
(a) The Process and the Company Context. Data from survey 3 (left). Participants and their commitment. Data from survey 3 (right). (b) Participants Feelings Regarding Process Identification and Innovation. Data from survey 2.

 In the three surveys conducted in November 2020, February 2021, and June 2021, survey participation was 53%, 75%, 45%. Relevant results from the three surveys are highlighted below in combination and shown in Figure 3.

###  Goals and Expectations

 Most employees (85%-90%) could closely identify with both the overall project and the goals their working groups were pursuing. Moreover, 70% of responders felt that they had served as agent of change and innovation, 80% felt actively involved in the project processes and in promoting positive changes in the hospital context (Figure 3a-3b).

 More than 80% of employees believed the project’s global goals were clear and specific enough from the start, while acknowledging the complexity of the hospital environment and the challenge of applying the project objectives to the hospital context: 95% of employees stated that goals were highly innovative for the hospital environment and more than 60% of employees stated that the goals were attainable (Figure 3a-3b).

###  Attendance and Effort

 Sixty percent (60%) of the staff stated they had dedicated lots of time and effort to the project; more than 70% who were also were satisfied by the balance between effort required and achieved project’s goals, reported that it was important this effort was sustained (Figure 3a-3b).

 Recommendations for improvement included a clear budget and specified deadlines. Respondents also emphasized the importance of getting a specific mandate and inputs on available resources at the start of a project.

###  Methods and Tools

 The support of the WHO team were unscored as both a valuable and crucial element during the project development. WHO’s contribution to facilitating and managing meetings, drafting minutes, introducing new methodologies, and assigning tasks were identified as important factors that fostered the work itself and the discussion and interaction amongst stakeholders.

 Employees noted a novel increase in cooperation between departments and the value of taking a multidisciplinary approach to topics.

###  Outcomes

 Moreover, the staff acquired or improved their skills in elements such as time management, organizational strategies, teamwork and problem solving. The participants reported an increasing ability to interact with other stakeholders in daily working routine and new ways of approaching tasks and projects.

###  Global Process

 During the survey, 99% of professionals involved in the process were willing to continue participating in the project in the future.

###  SWOT Analysis

 The flexible recruitment strategies and multidisciplinary approach ensured heterogeneity of the groups. However, recruitment processes were unstructured and not specified beforehand. Participants took part voluntarily and were recognized for their efforts. But this implied that only committed^14^ members facilitated the attainment of project goals. Having the hospital staff plan and execute the project ensured that the project was ingrained in the hospital’s day to day activities and limited the discrepancy between existing hospital and project activities and served as a suitable exit strategy for the external team. The project employed a mix of strategies and was closely monitored from its onset to initiate timely corrective and preventive actions. When defining areas of work or topics for strategic analysis, the dual approach – top down and bottom up – ensured inputs were relevant to multiple stakeholders.

 Furthermore, the presence of an external team of facilitators and established stakeholder relationships allowed the development of a collectively shared narrative thread and an increased cross communication amongst all actors involved. Due to a variety of departments being represented in the process, a multifaceted approach to topics was ensured (Box 1).

**Box 1. SWOT Analysis**

**Strengths**

- A mixed approached to topics: inputs from the Board of Hospital Directors + inputs from the groups using qualitative and quantitative data to inform the project
- The bottom-up approach allows to detect minor challenges that play a role considering the general hospital service quality
- The multidisciplinary approach and the support from external institutions allow to find non-standard solutions
- Constant monitoring of the process: keeping track of decisions, stakeholders involved, tasks and responsibilities using specific tools
- Presence on the external team in the field allowed for informal communication with the staff and in-person observation of facility structures and processes
- Flexibility in recruiting participants
- Internal professional recognition for participation
- Voluntary
- Building on established relationships or past experiences
- Team building and sense of belonging to the groups using team logos, gaining buy-in from the hospital head
- Enhanced communication between stakeholders: projects were approached through a multifaceted method

**Weaknesses**

- The absence of a structured format to participant recruitment. Need for clear ways of recruiting participants: roles and responsibilities, skills, expertise, commitments
- The lack of commitment by some stakeholders may slow the process and total absence of some stakeholders
- Need for a stronger mandate by the Board of Hospital Directors
- Issues in time scheduling: need for clear deadlines associated to projects
- Need for more structured surveys aimed at getting precise feedback from the staff
- Passive/voluntary participation & participation of the staff depending on Directors’ commitment
- Performance and talent management: need for a clearer framework of how to measure engagement, commitment, participation, achievements (as a group and individually)

**Opportunities**

- Interrelation with external stakeholders
- Integrating the project with the ordinary hospital hierarchy and decision processes
- Exportability/scalability of methods
- Cross communication and information exchange created through “the hospital of tomorrow” project influenced others hospital projects/hospital organizational aspects

**Threats**

- Discrepancy and gaps between the project and ordinary hospital processes
- Lack of broad communication towards the hospital staff
- Poor knowledge of the project by relevant stakeholders
- No clear projects approval or rejection during the presentations to board of directors could bring the groups to a waste of time on useless projects

--------------

Abbreviation: SWOT, Strengths, Weaknesses, Opportunities, and Threats.

## Discussion

 In this case study, it was evident that the mix of strategies and approaches promoted a conceptual analysis of all topics. Multidisciplinarity, heterogeneity and commitment, as evidenced by attendance were essential to the attainment of the project’s goals. The project was closely monitored to aide project performance. An increase in communication amongst all stakeholders was also observed over the project’s duration. The presence of an external team of facilitators and established relationships facilitated communication processes and fostered interrelation between all stakeholders involved.

###  Goal Attainment

 Goals setting was a crucial step of the global process clarity, efficacy and support in a first phase of goals’ identification foster the identification of professionals themselves and consequent engagement in the process.^23^ By joining the groups, the staff had the chance to set the focus on topics and goals whose essentiality and transversality were observed in the hospital environment by different departments and stakeholders. The multidisciplinarity of the groups allowed to consider multiple perspectives and work consequently. Nurturing interaction and a diverse environment promoted professional creativity and yielded innovative solutions. After a preliminary needs’ analysis, the staff worked to benchmark St. Orsola’s standards and procedures with national and international standards for potential importation of innovative inputs into the hospital in the short-term or long-term. Aligning a project’s goals to the overall institutional strategic objectives is essential to attainment of the project goals.^24^ Goal setting should incorporate aspects of integration with the hospital environment and parallel elements of innovation.

 Goals need to be SMART: Specific, Measurable, Achievable, Relevant, Time-based and at the same time they need to be shared and supported by all levels of the organization. That helps giving a clear direction to the related performance. The working groups set goals short-term and medium/long-term goals, including outcomes desired and time frame expected.^25^ Indeed, as project tasks began, the working groups identified which goals were feasible in the specified time frame. The working groups and task-forces that focused on shorter term goals easily received approval from the Board of Hospital Directors and was able to attain them. Conversely, extensive topics (traceability, wayfinding, telemedicine, etc) that required a longer time to implement and the involvement of many stakeholders were temporarily paused for further deliberations. There was only a two-level hierarchy within the project with working groups presenting goals to the Board of Directors; a strategy that fast-tracked decision making and limited bureaucratic steps. Project planning is essential to project success.^26^

###  Group Membership

 There was a high level of heterogeneity in job profiles, departments and levels involved in the process. Most projects require a skills-mix from a combination of experts; projects characterized by high levels of cross-functional cooperation have a higher chance of attaining project goals.^27^ Since distributive leadership is essential for the implementation and institutionalization of complex organizational changes,^28^ similar projects ought to incorporate through and accurate participant selection based on skills needed for each phase of the project. Additionally, a continuous process of participants’ recruitment would benefit such projects.

###  Strategies Employed

 The team used a combination of strategies to support the attainment of its goals. In educational projects, a combination of bureaucratic and normative cues to individual and institutional to action, incentive for participation and opportunities to acquire new skills are essential to a project’s success.^29^ In this project, strategic areas of intervention and working groups were created by the mandate of the Board of Directors and aims and ambitions of single professionals. Consequently, there was a more comprehensive analysis working areas and numerous proposals on how to address specific issues. When projects wish to create an efficient framework of talent and performance management within complex organizations, the objectives of individuals should correspond to those of the institution’s.

 This is because projects being only bottom-up might face a top-down reverse indication or being rejected due to budget or strategy constraints.

###  Project Timeline and Process Monitoring

 From the onset, the project team defined a project framework and scheduled milestones for rational allocation of time and resources. Furthermore, the staff were always cognizant of the next steps. These processes permitted the employees to focus on organization-relevant topics and limited the possibility of presenting unsuitable projects that would not be approved by the hospital board.

 By monitoring indicators of attendance, communication, goals’ setting and achievement and organizational impact, the WHO team detected major improvements in the way issues and priorities were approached by the staff, analyzed by working groups and brought to the attention of the Board of Directors. The case study shows the relevance of creating a triangulation between elements as scope, cost and time.^30^ As evidenced by the project’s development, the indicators monitored and the measured outcomes, the existence of one of these cannot be separated from the others: when starting a process as the one described, all these elements need to be considered and be clearly set both by the external team and all employees, so as for key pillars and assumptions to be openly shared.

###  Communication

 Communication processes are crucial throughout a project’s life cycle: a robust communication network should be created and cultivated amongst all actors. Cross functional teams that succeed differ from those that do not succeed based on their mode of communication (both formal and informal) and reasons for communication.^17^ The project set a focus on an increasing need for open communication amongst stakeholders who, over the months, attained several positive outcomes from the continuous interaction of network of job profiles, skills and hierarchy levels. Due to groups meeting regularly and professionals working cooperatively in taskforces, a fair number of the initially set goals were actually achieved. Furthermore, the necessary information about the resources available was communicated in time to support the hospital staff to set realistic contextual goals. Surveys are essential throughout a project’s life cycle.^31^ Such surveys fostered interaction amongst Departments and employees, with the team of external facilitators and illustrated a variety of challenges rooted in the hospital during project implementation. Surveys ought to be planned from the beginning to foster homogeneity, clarity, transparency and reliability of results. The questions posed ought to facilitate survey completion by employees and the collection and analysis of survey data that supports decision-making and allow potential readjustments or ad hoc actions to be taken.

###  Commitment

 The actual commitment of participants was critical at all points of process development. It was evident that the more the Department Directors were engaged in the process, the more their subordinates’ joined meetings and actively contributed to meeting discussions.

 The Board of the Hospital Directors gave very specific mandates to each Department and professional involved to foster a more participative process, where all stakeholders required were brought on board. While some professionals attended meetings and joined task forces regularly – and consequently brought their specific skills and competences –, some others tended to see it under a more voluntarist light and, by not joining frequently, leading to some gaps in the expertise required for projects’ implementation and temporary stand-still situations. There is a need for wholesome stakeholders’ participation to incorporate all skills and resources available in the process. Future projects should have the Board or team members set attendance and participation criteria for all members.

###  The Role of External Teams

 New projects may require different types of external resources, for instance, human resource, materials etc.^29^ External experts bring in ideas, focus and impetus to facilitate project delivery.^29^ The WHO team acted as a process facilitator by promoting continuity, consistency and suitable links between different phases of the project. The WHO team encouraged employees to focus on specific topics, maximize potential, avoid resources fragmentation and continue to progress. In addition, it proved crucial to the project’s final results to equip hospital staff with the skills and tools needed to navigate the project. Moreover, the WHO team acted as a liaison between stakeholders, fostering the formation of new networks and relationships, both within the organization and with third parties such as universities or other institutions. These network processes would benefit from the project from inputs, ideas and resources.

###  Project Ownership and Exit Strategy

 The Board of Hospital Directors was encouraged to gradually identify other stakeholders to take charge of supporting the process to avert the risk of resources or skills dispersion. They tasked supervisors or other persons involved for project sustainability. Person to whom specific tasks were assigned were invested in creating links with other employees or Departments whose contribution was needed to keep all persons involved up to date. At St. Orsola, two Boards of Innovation and Organizational Development were created to serve as a link between the Board of Hospital Directors, Focal Points and, consequently, working groups. Thus, promoting communication between different levels in the hospital hierarchy; an issue reiterated as critical during the surveys.

###  Limitations

 There were some limitations. Although we used an adaptable ad hoc participant recruitment strategy, it would have been beneficial to use a more systematic approach.

 The project’s timeline was not always easy to follow. Lack of clarity regarding the timeline and procedures for projects’ approval or rejection caused confusion and demotivation. This was primarily due to the fact that many initial projects were exclusively created using a bottom-up methodology without the Board’s input. Groups subsequently received precise directives with a deadline.

 The hospital staff initially perceived meeting attendance as additional work beyond what was required of them each day. Initially, the staff joined the project in accordance with their weekly availability.

 Areas of improvement is found in Risk Management: as mentioned, groups struggled in detecting stand-by situations or need for external inputs: this led to some lack of clarity in steps planning.^32^ When projects were not specifically assigned to managers in charge of their implementation, tasks were also not clear and groups struggled in detecting the right stakeholders to contact for specific contributions. Steps and stakeholders being defined at an early stage would allow processes to be smoother, responsibilities to be clear, risks to be circumscribed and solved and therefore operational steps to be planned more easily.

 Since budget lines and methods of its distribution amongst projects and stakeholders were not clarified at early stages, this led to stand-still situations where managers of Departments would not feel specifically in charge of using their portion of budget for projects’ implementation.

 Furthermore, monitoring and evaluation steps were pre-planned but implementation methods and related timelines were not defined.

## Conclusion

 The project’s success could be attributed to different factors. Key to the success of the project was the definition of time-bound goals that were both innovative and SMART for the hospital environment, after carefully analyzing the project context at the onset. The monitoring of the implementation with the possibility of adjustments as needed supports the achievement of the objectives. The persons supervising this project had the required skills/profiles from the project or the project network at all hierarchical levels. Management encouraged an environment of open communication at all levels before, during and after the project and ensured that the contributions of all members were taken into consideration. The commitment to the project’s success was exercised at all hierarchical levels, but above all by the Board of Directors. Despite involving the support of a team of external facilitators, a clear exit strategy ensured that the project would proceed after the departure of the external team.

## Acknowledgements

 The authors would like to thank all the IRCCS Azienda Ospedaliero-Universitaria di Bologna, Policlinico di Sant’Orsola staff and Board of Directors that supported and encouraged the implementation of the case study, KU Leuven University, Delft University of Technology, University of Padova and University of Bologna that contributed to the design of projects and WHO for the support on conducting and writing the case study.

## Ethical issues

 Not applicable.

## Competing interests

 Authors declare that they have no competing interests.

## Disclaimer

 The authors alone are responsible for the views expressed in this article and they do not necessarily represent the views, decisions or policies of the institutions with which they are affiliated.

## Authors’ contributions

 FG, AS, and AB conceived and designed the case study and were in charge of overall direction and planning of the project, wrote the manuscript with input from all authors. CG, LL, and MS conceived of the presented idea, encouraged the development of the case study, supervised the findings of this work, final approval of the version to be published. BB aided in interpreting the results, writing of the manuscript in consultation with AS, AB, and FG. JR and LF conceived the original idea, was involved in planning and supervising the work, contributed to the design and implementation of the project.
